# Communicating with people living with dementia who are nonverbal: The creation of Adaptive Interaction

**DOI:** 10.1371/journal.pone.0180395

**Published:** 2017-08-01

**Authors:** Maggie Ellis, Arlene Astell

**Affiliations:** 1 School of Psychology and Neuroscience, University of St Andrews, St. Andrews, United Kingdom; 2 ScHARR, University of Sheffield, Sheffield, United Kingdom; 3 Research and Academics, Ontario Shores Centre for Mental Health Sciences, Whitby, Canada; University of Ottawa, CANADA

## Abstract

Loss of verbal language production makes people with dementia appear unreachable. We previously presented a case study applying nonverbal communication techniques with a lady with dementia who could no longer speak, which we termed Adaptive Interaction. The current small-n study examines the applicability of Adaptive Interaction as a general tool for uncovering the communication repertoires of non-verbal individuals living with dementia. Communicative responses of 30 interaction sessions were coded and analysed in two conditions: Standard (Baseline) and Adaptive Interaction (Intervention). All participants retained the ability to interact plus a unique communication repertoire comprising a variety of nonverbal components, spanning eye gaze, emotion expression, and movement. In comparison to Baseline sessions, Intervention sessions were characterised by more smiling, looking at ME and imitation behaviour from the people with dementia. These findings allude to the potential of Adaptive Interaction as the basis for interacting with people living with dementia who can no longer speak.

## Introduction

Dementia is an umbrella term used to describe a collection of diseases characterised by progressive loss of cognitive functions and other abilities. Dementia has a neurological basis that is typically untreatable and irreversible [1], and the greatest risk factor for developing it is age [1]. People who are living with dementia typically experience gradual but insidious decline in a wide range of abilities over a number of years [2], which may ultimately result in them relying on family or formal caregivers to meet all of their needs [3]. As the illness progresses to the later stages, care is often provided in nursing homes or other institutions. As such, staff members need to form relationships and get to know people who already have significant communication difficulties by the time they meet [4].

As dementia progresses conversation becomes increasingly challenging and towards the later stages, verbal language production may disappear altogether [5]. People living with dementia may make nonverbal attempts to communicate with caregivers but these are typically ignored [6], misinterpreted as ‘challenging’ [7] or judged incomprehensible [8]. The communicative difficulties experienced by people with dementia are not only misinterpreted as signifying that they have nothing to contribute, but that they have actually lost the desire to participate in the social world [9]. This perception manifests in low levels of reported social activity by people with dementia in residential care. For example, Bowie and Mountain (1993) [10] observed 110 people with dementia living in a long stay hospital ward, who they found spent only 5.5% of their day involved in social engagement but 68% in neutral, i.e. no activity. Furthermore, the authors noted that the majority of social engagement occurred during caregivers’ pursuance of basic activities of daily living [10]. In other words, people with dementia were only given the opportunity to engage in social interactions when they were being helped to eat or assisted with personal care. Consequently, individuals with advanced dementia often find themselves excluded from the social world and negated as social agents [11], [6].

A lack of social interaction not only leads people with dementia to withdraw from social life [6], it also has a negative impact on caregivers [12]. This may reflect the tendency of caregivers of people with dementia to view verbal language production as an indicator of ‘emotional connection’ [13], making those without verbal language production appear ‘unreachable’. Thus when faced with someone who has lost the ability to speak, caregivers often withdraw from those they care for. This detachment may be due to discomfort on the part of caregivers who distance themselves as a method of coping with the demands of the situation [14]. Several studies have reported a relationship between poor attitudes towards people with dementia and accounts of high ‘burnout’ in care staff [15], [16], [17], [18]. A lack of motivation and education among staff can lead to reduced levels of staff-resident interactions as staff feel unable and/or unsupported to communicate with people with dementia [19], [20]. As such, finding alternative methods of communication in the care environment has the potential to improve not only the quality of life of people with advanced dementia, but also the job satisfaction of care staff [21]. The challenge is therefore to provide caregivers of people living with dementia who can no longer speak, with the means to keep interacting and communicating.

We have previously demonstrated that using an approach based around the nonverbal aspects of communication, i.e. sounds, movements, facial expressions, etc.—has the potential to keep people with advanced dementia in the social world [22], [23]. In this single case study we explored the utility of Intensive Interaction [24], [25] for communicating with Edie, an 83-year old lady with very advanced dementia, who was nonverbal and spent most of her days in bed alone in her room. Intensive Interaction is an approach developed initially to promote communication with people with severe and profound learning disabilities. It is based on the fundamentals of communication that typically accompany language acquisition in early life, including eye gaze, emotional expression and movements. The focus of Intensive Interaction is on learning the communicative repertoire of an individual who is nonverbal through observation and imitation. These behaviours are thought to represent ‘self-talk’ on the part of individuals with severe and profound learning disabilities that become the basis of an interaction when mirrored by a communication partner. For example, the caregiver might copy a sound or action made by the nonverbal person, such as tapping the table. Through attending to the other person’s behaviour and responding contingently, the caregiver is able to expand the interaction and support her partner to take a more active role in communication. By responding in ways that are familiar and meaningful to the person with severe communication difficulties, i.e. initially imitating and then developing them into a shared ‘language’, it is possible to build and sustain close relationships without verbal language production [26]. Interactions are developed on a day-to-day basis as interaction partners become increasingly attuned to each other and develop their shared language.

In our work with Edie, we found that applying the principles of Intensive Interaction uncovered her nonverbal ‘language’. This comprised a rich repertoire of sounds, including laughter, eye gaze, movements, and facial expressions [23]. In comparison to a Baseline interaction comprising closed questions of the type observed to be used by the caregivers in the nursing home where Edie lived, Intensive Interaction revealed Edie to be a responsive interaction partner, who was able to turn take and even take the lead. For example, Edie moved her head to the side of the bed where the investigator’s hand was resting. After several attempts she succeeded in just touching the investigator’s hand. When the investigator responded by bringing her own head down to gently touch Edie’s, Edie’s eyes flew wide open in a look of surprise. Her gaze then locked onto the investigator and they made a sound towards each other until they both start to laugh [23] (see S1 File. Example of Adaptive Interaction, for extended account of this interaction).

In order to respond to the communication needs of people with advanced dementia some modification of Intensive Interaction was required. Specifically, due to the severe memory problems experienced by people with dementia, Intensive Interaction with this population must remain ‘in the moment’ with no need for any parts of previous interactions to be remembered. Therefore, the communication partner must remain ‘adaptive’ to the changes in communication by the person with dementia and be willing to start afresh each time. As such, we named the approach Adaptive Interaction.

Building on the case study the aim of the present study was to investigate whether Adaptive Interaction can be used as a general tool for uncovering non speech-based communication repertoires in other people living with dementia who can no longer speak. To address this participants were video recorded in both Standard (Baseline) and Intervention (Adaptive Interaction) sessions and their behaviour in both coded using a scheme based on the fundamentals of communication developed in the case study.

## Method

### Design

The experimental design selected for the present study was an AB Multiple Baseline design in which the Baselines represent the multiple participants. This design consists of 2 phases (Baseline and Intervention) and Intervention points are randomly allocated to the participants. With 6 sessions for each participant, and a minimum of 2 sessions of each phase (i.e. 2 Baseline and 2 Intervention), there were 3 possible intervention points in each stage. Thus, participants were randomly assigned a starting point for the Intervention, occurring anywhere between session 3 and session 5, ensuring that there was a minimum of 2 sessions of baseline (A) and 2 sessions of Intervention (B).

The randomisation of Intervention starting points for each dyad ensures internal validity in the design by controlling for the effects of exposure to sessions and increased attention as possible explanations for changes in behaviour. By randomising the starting points for the Intervention, it is possible to determine whether exposure to the Intervention has an effect on the behaviour of participants. If exposure to sessions had an effect on behaviour, then it would be expected that there would be similar shifts in the behaviour of each participant, regardless of when the Intervention was introduced. If, however, there was little effect of exposure to the Intervention, then the most significant changes would be expected to take place at the point at which the Intervention had been introduced. Thus, noticeable shifts in behaviour at Intervention points are likely to indicate a main effect of Intervention. Table 1 shows the running order of Baseline and Intervention sessions for all participants.

**Table 1:** The running order of Baseline and Intervention sessions for all participants.

Participant	Session number	Session type
1	1	Baseline
	2	Baseline
	3	Intervention
	4	Intervention
	5	Intervention
	6	Intervention
2	1	Baseline
	2	Baseline
	3	Baseline
	4	Intervention
	5	Intervention
	6	Intervention
3	1	Baseline
	2	Baseline
	3	Baseline
	4	Intervention
	5	Intervention
	6	Intervention
4	1	Baseline
	2	Baseline
	3	Baseline
	4	Baseline
	5	Intervention
	6	Intervention
5	1	Baseline
	2	Baseline
	3	Baseline
	4	Intervention
	5	Intervention
	6	Intervention

### Participants

Five participants (1 man) living with dementia ranging in age from 77–89 years (mean 82.6 years) took part in the study. The participants all lived in the same nursing home and had been resident for at least three years. The nursing home manager was asked to identify residents who had a diagnosis of dementia or probable Alzheimer’s disease and who had very little or no retained verbal language production. Only one of the five participants was independently mobile, one participant spent most of the day in the sitting room in her wheelchair and three were largely confined to bed for most of the day.

At 89 years old participant 1 (MB) was the eldest and the only participant who was independently mobile. She spent most of the day walking up and down the corridors but was helped to her room to relax at several points during the daytime. She also had the most verbal language production of all the participants (approximately 10 words) and was eager to communicate. Her daughter worked as a domestic assistant at the home and as such she was able to see her most days.

Participant 2 (MD) was 88 years and 8 months old and a wheelchair user. Although she had some verbal language production, most was composed of speech-like sounds that were difficult to decipher. She spent most of her time either in the day room or her own room and was willing to communicate when approached. Her daughter visited her regularly and she often took her mother to the day room in her wheelchair.

Participant 3 (BS) was 81 years and 7 months old and confined to bed. He was extremely fragile and had to be hoisted to and from bed and as such only went to the day room or to the dining room when taken by staff members. He was very quiet but made speech-like sounds and formed some words when encouraged. His brother and sister-in-law visited him from time to time but he had no regular visitors.

Participant 4 (EA) was 81 years old and although she had no discernible verbal language production she made a high-pitched sound that she used frequently, particularly when someone entered her room, where she spent large amounts of time in bed. Her daughter visited daily but she was not often taken to the day room as her sound allegedly “disturbed” the other residents. As such, EA only had the opportunity to engage with other people when someone came to her room.

Participant 5 (GB) was 77 years and 11 months old, was largely confined to bed but was taken to the dining room for meals in a padded chair. GB was extremely quiet and had no discernible verbal language production. She had very few visits from her family and spent most of her time alone in her room.

### Materials

The ‘Direct Observation of Behaviour’ (DOB; Bowie and Mountain, 1993 [10]) is an observation tool developed to provide an assessment of the amount of time people with dementia in residential care settings are engaged in different types of behaviour during the day. The DOB comprises seven categories in which the description of behaviours in the first three categories (i.e. ‘self-care’, ‘social engagement’ and ‘reception of care’) use adjectives that suggest positive behaviours (i.e. ‘independent’, ‘purposeful’, ‘appropriately’, ‘actively’, ‘cared for’) that might be regarded as ‘acceptable’ or ‘typical’. Conversely, the remaining four categories (i.e. ‘motor activity’, ‘antisocial’, ‘inappropriate’, ‘neutral’) use more negative descriptors (i.e. ‘unnecessary’, ‘excessive’, ‘aimless’, ‘violate’, ‘cause distress’, ‘aggression’, ‘unacceptable’, ‘inappropriately’, ‘detached’), suggesting behaviours that may appear to be meaningless and/or that caregivers might find ‘challenging’.

A Sony Mini DV Video camera and tripod were used to video and audio record all sessions. Timing was provided by a mobile phone.

### Protocol

#### Ethics and consent

Ethical approval for the study was received from the Scotland MREC Committee A, that deals with research proposals covered by Section 51 (3) (f) of the Adults with Incapacity (Scotland) Act 2001, i.e. research with people who are unable to give informed consent. In accordance with the AWI (Scotland) Act 2001, consent was sought from the nearest family member for the people with advanced dementia to participate. The approval included permission to video and audio record the interactions.

The protocol involved two stages:

Stage 1: Observation of daily activities/familiarisation.

**Fig 1:**
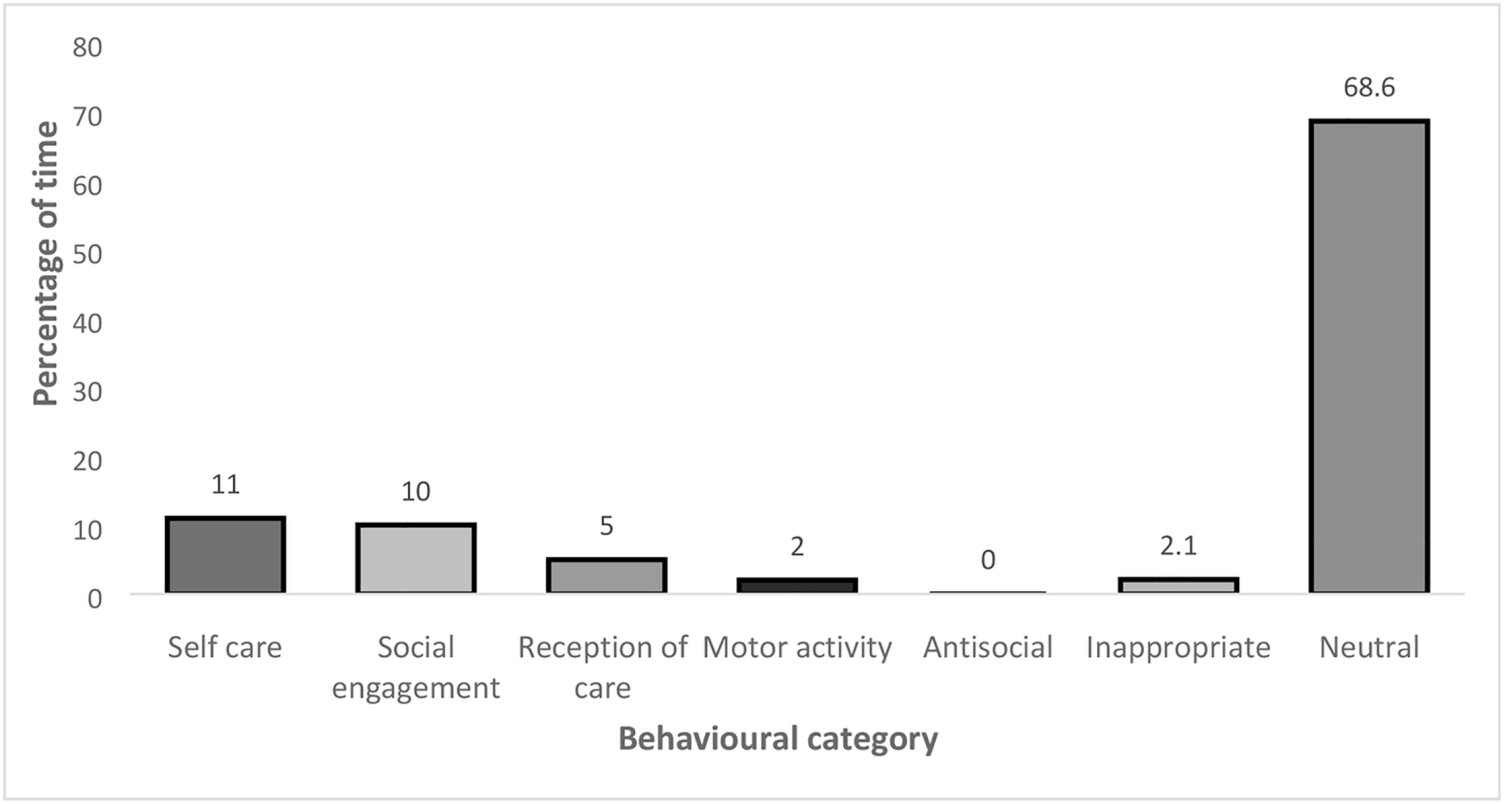
The percentages of each behavioural category for all participants recorded over two days.

The first stage involved two observers using the DOB [10] over two days to collect data on the activities and interaction patterns of the participants and to build up a picture of their daily routine. The observers were the first author (ME) and a trained senior honours student, neither of whom had any relation to the participants or to their care. Each participant was observed for one minute every ten minutes and a decision made about what behaviour they were performing using the behaviour categories defined by Bowie and Mountain [10] (1993; Fig 1). The categorisation of behaviours was agreed 100% by both observers. Participants were observed between 10am and 4pm on both days. This phase was crucial for taking field notes regarding the communication environment and providing insight into the opportunities available for, and occurrence of, social interactions.

Stage 2: Interaction

Stage 2 involved two conditions:

1. Standard Interaction (Baseline condition).

This was a verbal language production-based interaction where ME spoke to the person with advanced dementia using closed questions such as “Did you enjoy your meal?”, “Did you have a lie in this morning?” and “Have you seen the weather outside today?” (See S2 File. Baseline interaction questions, for full list). The rationale for using these types of questions is as follows. Firstly, as this was a ‘Baseline’ interaction it was important that ME used a form of communication that was closely related to that used by those who interacted most with each participant, i.e. professional caregivers. Observers noted in Stage 1 (Observation of daily activities/familiarisation) that these were the types of questions used to communicate with participants. The second reason for using closed questions, such as “Have you seen the weather outside today?” was that for those participants who understood the question but who were unable to answer using verbal language were given the opportunity to respond non-verbally (by nodding or shaking their heads). By comparison, open questions involving lexical search, e.g. “How do you think the weather looks outside today?” do not provide participants with the opportunity to respond non-verbally if they understand the question but verbal language is not accessible to them. The use of closed questions is widely recommended as a method of simplifying language for individuals with dementia who have problems with open questions.

2. Adaptive Interaction (Intervention condition).

This was a nonverbal interaction whereby ME attempted to communicate with the participants using their own sound-based and non-verbal behaviours as the basis of interaction [26] (See S1 File. Example of Adaptive Interaction 1, for further information).

Both Standard and Intervention conditions were conducted by the ME (Author 1) in order to provide consistency throughout the sessions.

### Procedure

The closest family members of the participants were contacted via the nursing home and provided with all information on the study. The family members were asked to give consent for ME to approach their relative with advanced dementia. Family caregivers were asked to give proxy consent for their relatives to take part and were also invited to be present when the study took place. Where this was not possible, members of care staff were invited to observe the sessions to facilitate the recognition of any signs of distress in the individual participants, which would lead to immediate cessation of the sessions. In the event, neither family nor care staff elected to observe any of the sessions. As such, all participants engaged with ME in the absence of any other people.

A timetable of sessions was agreed with the nursing home manager to minimise interruption to the participants’ daily routines. As such, sessions were organised to take place between 10 am and 12.30 pm (between breakfast and lunchtime) and 1.30 pm and 4 pm (between lunch and dinnertime). Each session was planned to last for a maximum of ten minutes. Table 1 illustrates a timetable of sessions for each participant. All sessions were video recorded and participants were filmed interacting with ME in a total of six sessions comprising a mixture of both Baseline and Intervention. No sessions were terminated through participants presenting visible signs of distress. Seven sessions were interrupted for the following reasons: participant fell asleep during session (n = 5); participant unwell (n = 1); participant stopped interacting (n = 1).

#### Coding of communicative behaviours

Three minutes from the beginning of each clip was selected for coding so as to eliminate the majority of the loss of data from sessions that were interrupted. Microanalytic coding captures intricate detail of behaviours second-by-second. Coding categories were developed from those identified in the case study [23] and the observation/familiarisation phase of the current study. These were supplemented by examination of categories employed in studies of Intensive Interaction in individuals with learning disabilities [27], [28], [29]. This yielded the following six coding categories: 1. Eye gaze; 2. Facial expression; 3. Vocalisations; 4. Gesture; 5. Physical contact; and 6. Imitation. Table 2 shows the main behavioural coding categories and subvariables.

**Table 2:** The main behavioural coding categories and subvariables.

Coding category	Subvariable
1. Eye gaze	Eyes closed
	Looking at ME’s face or body
	Looking elsewhere
	Can’t tell
2. Facial expressions	Neutral
	Smiling
	Frowning
	Surprise
3. Vocalisations	Vocalisation
	Laughter
	Silence
	Other (e.g. coughing)
4. Gestures	Pointing
	Nodding
	Shaking head
	Other
5. Physical contact	Contact occurs
	No contact
	Can’t tell
6. Imitation	Person with dementia imitates ME

The Observer^TM^ Pro version 5 was used to conduct the behavioural coding [30].

#### Inter-rater reliability

ME coded 100% of the three-minute segments of the 30 sessions (6 per participant) for verbal and non-verbal communicative behaviours. Four sessions (13.3% of the total amount of sessions) were selected at random and were coded by a second rater (trained senior honours student). It was not possible for either rater to be blind to the study and/or the participants as the differences between the conditions and participants were apparent when conducting behavioural coding of video and audio output. Kappa values were calculated for 4 sessions coded by both raters. Landis and Koch (1977) [31] suggested that a kappa value of equal to or less than 0.20 indicates slight agreement; 0.21–0.40, fair agreement; 0.41–0.60, moderate agreement; 0.61–0.80, substantial agreement; and 0.81–1.00, almost perfect agreement. The Cohen’s kappa values were calculated as follows: session 1 (Participant 4/Baseline): 0.91; session 2 (Participant 1/Intervention): 0.58; session 3 (Participant 5/Intervention): 0.80; session 4: (Participant 3/Baseline): 0.59, indicating that the interrater reliability ranged from moderate to almost perfect agreement.

### Data analysis

#### Direct observation of behaviour [10]

The percentage of time that each participant was engaged in each of the seven categories of behaviour on the DOB [10] was calculated. These data were pooled to provide the total percentages of time all five participants cumulatively spent engaged in each type of behaviour.

#### Individual communication repertoires

The data for each participant were examined for the presence of communicative behaviours that could be said to comprise that individual’s communication repertoire. This was based on the behavioural coding analysis described above (Table 2).

#### Group comparison

A second stage of analysis involved pooling the data from the five participants across the 15 Baseline and 15 Intervention sessions to compare the frequency and/or duration of occurrence of behaviours in the six coding categories (Table 2). This was to examine the effect of the Intervention on the occurrence of communicative behaviour by people living with dementia who can no longer speak.

## Results

The data are presented in three parts: 1. Direct Observation of Behaviour [10] for the five participants, 2. Individual communication repertoires, and 3. Group comparisons.

### 1. Direct observation of behaviour [10]

The observation data (100% agreement between observers) revealed that over two thirds of the participants’ time (68.6%) was spent in neutral activity, that is ‘detached from the environment’, typically sleeping or doing nothing. The total percentage of time coded as the other negative behaviour categories (‘motor activity’, ‘antisocial’ and ‘inappropriate’) was extremely low at 4.2% (Fig 1). Fig 1 shows the percentages of each behavioural category for all participants recorded over two days.

### 2. Individual communication repertoires

Each individual’s communicative vocabulary could be coded under broad categories, such as eye gaze, movements, vocalisations, etc. however, as one might expect, there was some variation of behaviours within those categories. As such, a coding scheme was created for each participant defining his/her individual communicative behaviours. Table 3 lists the communicative behaviours that appear for each individual. The frequency of these behaviours was logged across all of the sessions. Table 3 illustrates the sub-behaviours under each main category engaged in by each participant.

**Table 3:** Communication modalities and sub-behaviours identified for each participant across both Baseline and Adaptive Interaction sessions.

Participant						
Modality	Behaviour	1 (MB)	2 (MD)	3 (BS)	4 (EA)	5 (GB)
Eye gaze	Looking at ME					
	Eyes closed					
	Eyes closed tight					
	Looking at camera					
Sound	Coughing					
	Yawning					
	Speech sounds					
	Clearing throat					
	Heavy breathing					
	Laughing					
	High pitched sound					
	Growling sound					
	Clicking tongue					
	Sighing					
	Singing					
	Tutting					
Movement	Pointing					
	Nodding					
	Shaking head					
	Finger in/on mouth					
	Looks at/plays with hands					
	Lifting head off bed					
	Poking out tongue					
	Drawing attention					
	Shrugging shoulders					
	Touching ME					
	Scratching chin					
	Wringing hands					
	Chewing thumb					
	Rubs head on bed					
	Rubs head on I’s hand					
	Rubs head on I’s head					
	Shaking body					
	Leaning forward to cam					
	Clasping & shaking hands					
	Rubbing knees					
	Scratching nose					
	Stroking chin					
	Feeling collar					
	Head side to side					
	Licking lips					
	Leaning into ME					
Facial expression	Eyebrow flash					
	Eyebrow raise					
	Smile					
	Frown					
	Surprise					
	Blowing kiss					
	Winking					
Total communicative behaviours person		30	13	9	10	10

As seen in Table 3. participant 1 engaged in 30 communicative behaviours in total (3/10% eye gaze; 9/30% sound; 13/43.3% movement; 5/16.7% facial expression); Participant 2 engaged in 13 communicative behaviours in total (1/7.1% eye gaze; 2/15.4% sound; 7/53.8% movement; 3/23% facial expression); Participant 3 engaged in 9 communicative behaviours (1/11.1% eye gaze; 4/44.4% sound; 2/22.2% movement; 2/22.2% facial expression); Participant 4 engaged in 10 communicative behaviours (2/20% eye gaze; 2/20% sound; 4/40% movement; 2/20% facial expression); Participant 5 engaged in 10 communicative behaviours (3/30% eye gaze; 3/30% sound; 3/30% movement; 1/10% facial expression).

It is clear (Table 3) that there was great variation in the types of behaviours for each participant within each of the behavioural categories. Indeed, the only behaviour that every participant engaged in was ‘looking at ME’. ‘Speech sounds’ and ‘smiling’ were engaged in by 4 of the participants. Three participants ‘closed their eyes’, ‘coughed’, ‘laughed’ or ‘pointed’. Two participants engaged in ‘nodding’, ‘shaking head’ and ‘frowning’. All other behaviours although categorised under the broader categories of ‘eye gaze’, ‘sound’, ‘movement’ and ‘facial expression’ were specific to each individual. For example, participant 1’s ‘leaning forward to camera’, participant 2’s ‘drawing attention’, participant 3’s ‘eyebrow flash’, participant 4’s ‘high pitched sound’ and participant 5’s ‘looking at/playing with hands’.

Tables 4–8 illustrate examples of each of the participant’s communicative behaviours and how many times they occurred during each minute and in total during randomly selected, 10 minute Baseline and Intervention sessions (1 of each).

**Table 4:** Number of communicative behaviours per minute between Baseline and Intervention sessions for participant 1.

Category	Minute	1	2	3	4	5	6	7	8	9	10	Total
**Standard interaction**												
Eyes	Looking at ME	4	4	3	5	3	2	5	4	2	3	35
	Eyes closed	1	2	6	3	5	5	1	4	1	2	30
	Looking at camera	5	6	6	3	6	1	0	0	0	0	27
Sound	Laughter	1	3	0	0	0	0	0	0	0	0	4
	Speech sound	1	3	3	6	3	7	4	6	5	5	43
	Growling sound	6	9	8	8	11	13	9	11	11	13	99
	Clicking tongue	0	0	0	8	10	0	0	0	0	0	18
	Singing	0	0	0	0	0	0	0	0	0	0	0
	Tutting	0	0	0	0	0	0	0	0	0	0	0
Movement	Nodding	3	1	4	3	2	1	3	3	2	1	23
	Shaking head	5	5	6	5	1	4	4	3	2	4	39
	Head side to side	1	0	1	0	0	0	0	0	0	0	2
	Shaking body	1	0	0	0	0	0	0	0	0	0	1
	Leaning forward to cam	2	1	0	0	0	0	0	0	0	0	3
	Clasping & shaking hands	0	3	0	0	0	0	0	0	0	0	3
	Rubbing her knees	0	0	0	0	0	0	0	0	0	0	0
	Scratching her nose	0	0	0	0	0	0	0	0	0	0	0
	Stroking her chin	0	0	0	0	0	0	0	0	1	0	1
	Feeling her collar	0	0	0	0	0	0	0	0	0	1	0
	Pointing	0	0	0	0	0	0	0	0	0	0	0
	Head side to side	0	0	0	0	0	0	0	0	0	0	0
	Licking lips	1	2	0	0	0	0	1	0	0	0	3
	Leaning into ME	0	0	0	0	0	0	0	0	0	0	0
Facial expressions	Blowing kiss	2	1	0	0	0	0	0	0	0	0	3
	Smiling	1	1	0	0	0	0	0	0	0	0	2
	Winking	1	1	1	1	1	0	0	0	0	0	5
	Frowning	1	0	0	0	0	0	0	0	0	0	1
	Raising eyebrows	0	1	0	0	1	0	0	0	1	0	3
**Adaptive interaction**												
Eyes	Looking at ME	2	3	0	1	0	0	2	0	2	1	11
	Eyes closed	0	0	0	1	2	1	2	1	3	5	15
	Looking at camera	3	4	1	3	2	0	0	0	0	1	14
Sound	Laughter	0	0	0	0	0	0	0	0	0	0	0
	Speech sound	3	4	8	1	0	1	0	1	1	3	22
	Growling sound	0	1	0	6	8	8	11	10	14	9	67
	Clicking tongue	0	0	0	0	0	0	0	0	0	0	0
	Singing	0	0	0	3	5	3	0	0	0	0	11
	Tutting	1	0	0	0	0	0	0	0	0	0	1
Movement	Nodding	0	0	0	1	0	0	0	0	1	0	2
	Shaking head	0	0	0	0	0	0	0	0	0	2	2
	Head side to side	0	0	1	1	2	1	0	0	0	1	6
	Shaking body	1	1	0	0	0	1	0	0	0	0	3
	Leaning forward to cam	0	0	0	0	0	0	0	0	0	0	0
	Clasping & shaking hands	1	2	1	0	0	0	0	0	0	0	4
	Rubbing her knees	0	0	0	0	0	0	0	1	2	0	0
	Scratching her nose	0	0	0	0	0	0	1	2	0	0	3
	Stroking her chin	0	0	0	1	0	0	0	0	0	1	2
	Feeling her collar	0	0	0	0	0	0	0	0	0	0	0
	Pointing	0	2	0	0	0	0	0	0	0	0	0
	Head side to side	0	2	0	0	0	0	0	0	0	0	2
	Licking lips	0	0	2	1	0	0	0	0	0	0	3
	Leaning into ME	0	0	0	0	0	0	1	0	0	0	1
Facial expressions	Blowing kiss	0	0	0	0	0	0	0	0	1	1	2
	Smiling	0	2	0	0	0	0	0	0	0	0	2
	Winking	0	1	1	0	1	0	0	0	0	0	3
	Frowning	0	0	3	1	0	0	0	0	0	0	4
	Raising eyebrows	2	1	0	0	0	0	0	0	0	0	3

**Table 5:** Number of communicative behaviours per minute between Baseline and Intervention sessions for participant 2.

Category	Minute	1	2	3	4	5	6	7	8	9	10	Total
**Standard interaction**												
Eyes	Looking at ME	3	4	3	2	2	1	0	0	1	3	19
Sounds	Speech sounds	4	6	4	5	5	5	3	7	5	4	48
	Laughing	2	0	0	0	0	0	0	0	0	0	2
Movement	Pointing	1	1	0	1	0	1	0	0	0	1	5
	Nodding	4	2	3	1	3	2	2	1	2	3	23
	Shaking head	1	0	2	0	0	0	0	0	0	0	3
	Drawing attention	0	0	0	0	0	0	0	0	0	0	0
	Shrugging shoulders	0	0	1	0	0	0	0	0	0	0	1
	Touching ME’s chin	0	0	0	0	0	0	0	0	0	0	0
	Moving hands	0	0	1	2	1	1	0	2	0	1	8
	Scratching chin	0	0	0	0	0	0	0	1	0	0	1
	Body jerk	1	0	0	0	1	1	0	0	1	0	4
Facial expressions	Frown	1	1	1	1	1	0	0	0	0	0	5
	Smile	1	0	0	0	0	0	0	0	0	0	1
**Adaptive interaction**												
Eyes	Looking at ME	2	2	2	2	2	3	2	2	3	1	21
Sounds	Speech sounds	1	3	2	3	4	1	6	7	2	4	33
	Laughing	3	0	3	1	1	4	3	0	4	1	20
Movement	Pointing	0	0	0	0	0	0	0	0	0	0	0
	Nodding	1	3	2	3	3	3	0	2	0	0	17
	Shaking head	0	0	0	1	0	1	0	1	0	0	3
	Drawing attention	0	0	1	0	0	0	0	0	1	0	2
	Shrugging shoulders	0	0	0	0	0	0	0	0	0	0	0
	Touching ME’s chin	0	0	0	0	0	0	1	0	1	0	2
	Moving hands	4	4	2	4	4	3	4	3	3	3	34
	Scratching chin	1	0	0	0	0	0	1	0	0	0	2
	Body jerk	1	0	1	0	0	0	1	0	0	1	4
Facial expressions	Frown	2	1	0	0	0	1	1	1	1	0	7
	Smile	3	1	4	2	1	3	3	0	2	1	20

**Table 6:** Number of communicative behaviours per minute between Baseline and Intervention sessions for participant 3.

Category	Minute	1	2	3	4	5	6	7	8	9	10	Total
**Standard interaction**												
Eyes	Looking at ME	6	5	6	1	5	3	2	3	5	3	39
Sound	Coughing	0	1	3	0	0	1	1	0	0	0	6
	Yawning	0	0	0	0	1	1	0	0	0	0	2
	Speech sounds	3	4	2	5	3	3	4	5	1	9	39
	Clearing throat	2	2	3	0	1	0	0	0	0	1	9
Movement	Pointing	1	0	0	0	0	0	0	0	0	0	1
	Hand in air	0	3	2	0	0	0	0	0	0	0	5
	Body jerk	1	3	3	3	2	1	3	1	1	0	18
	Finger in/on mouth	1	0	1	0	0	0	0	0	0	0	2
Facial expressions	Eyebrow flash	0	3	0	0	0	1	1	0	3	1	9
	Smile	0	0	0	1	0	0	0	0	0	0	1
**Adaptive interaction**												
Eyes	Looking at ME	5	4	7	3	3	3	3	3	2	2	35
Sound	Coughing	0	0	0	0	0	0	0	0	0	0	0
	Yawning	0	0	0	0	0	0	0	0	0	0	0
	Speech sounds	10	7	8	9	9	12	6	2	1	5	69
	Clearing throat	1	0	4	0	1	0	0	0	0	0	6
Movement	Pointing	6	4	9	8	6	7	2	1	1	0	44
	Hand in air	3	3	1	1	0	2	1	0	0	0	11
	Body jerk	1	0	0	0	0	0	2	2	1	1	7
	Finger in/on mouth	3	1	0	1	0	0	0	0	0	0	5
Facial expressions	Eyebrow flash	0	1	2	1	0	3	1	1	1	0	10
	Smile	0	0	0	0	0	0	0	0	0	0	0

**Table 7:** Number of communicative behaviours per minute between Baseline and Intervention sessions for participant 4.

Category	Minute	1	2	3	4	5	6	7	8	9	10	Total
**Standard interaction**												
Eyes	Looking at ME	2	1	1	0	0	2	3	1	0	0	10
	Eyes closed	0	1	0	0	1	1	2	2	1	0	8
Sound	Coughing	0	0	0	0	0	0	0	0	0	0	0
	Yawning	0	0	0	0	0	0	0	0	0	0	0
	High pitched sound	0	1	2	0	1	3	3	1	2	2	15
	Laughing	0	0	0	0	0	0	0	0	0	0	0
Movement	Chewing thumb	1	0	0	2	1	2	1	3	4	1	15
	Rubs head against bed	0	0	0	0	0	0	0	0	0	0	0
	Rubs head against ME’s hand	0	0	0	0	0	0	0	0	0	0	0
	Rubs head against ME’s head	0	0	0	0	0	0	0	0	0	0	0
Facial expressions	Smile	0	0	0	0	0	0	0	0	0	0	0
	Surprise	0	0	0	0	0	0	0	0	0	0	0
**Adaptive interaction**												
Eyes	Looking at ME	1	1	1	2	1	1	2	2	1	1	13
	Eyes closed	0	0	1	1	0	0	1	1	0	1	5
Sound	Coughing	0	0	0	0	0	0	0	0	0	0	0
	Yawning	0	0	0	0	0	0	0	0	0	0	0
	High pitched sound	10	15	18	9	17	18	15	5	13	0	120
	Laughing	0	0	0	1	3	1	2	1	1	0	9
Movement	Chewing thumb	2	3	0	0	0	0	0	0	0	0	5
	Rubs head against bed	1	1	2	0	0	1	0	1	0	0	6
	Rubs head against ME’s hand	0	0	2	7	0	0	0	0	0	0	9
	Rubs head against ME’s head	0	0	0	2	5	5	3	0	4	0	19
Facial expressions	Smile	0	0	0	1	3	1	2	1	1	0	9
	Surprise	0	0	0	1	0	0	1	1	0	0	3

**Table 8:** Number of communicative behaviours per minute between Baseline and Intervention sessions for participant 5.

Category	Minute	1	2	3	4	5	6	7	8	9	10	Total
**Standard interaction**												
Eyes	Looking at ME	1	3	2	2	2	3	1	1	1	1	17
	Eyes closed tight	0	0	0	0	0	1	1	0	0	0	2
	Eyes closed	0	0	0	0	0	0	0	0	2	2	4
Sound	Coughing	0	0	0	0	1	0	0	0	0	0	1
	Speech sounds	0	0	0	0	0	0	0	0	0	0	0
	Heavy breathing	0	0	0	0	0	0	0	0	0	0	0
Movement	Looking at/playing with hands	1	1	1	1	1	3	1	2	3	2	16
	Lifting head off bed	1	0	2	0	4	2	0	1	1	0	11
	Body jerk	0	1	0	3	1	1	0	3	1	0	10
	Poking out tongue	0	0	0	0	0	0	0	0	0	0	0
Facial expressions	Raising eyebrows	3	2	0	2	3	4	3	3	1	1	22
**Adaptive interaction**												
Eyes	Looking at ME	4	5	6	8	5	2	1	2	4	4	41
	Eyes closed tight	0	0	0	0	0	0	0	0	0	0	0
	Eyes closed	0	0	0	0	0	0	0	0	0	0	0
Sound	Coughing	0	0	0	0	0	0	0	0	0	0	0
	Speech sounds	3	5	3	2	5	1	2	1	2	5	29
	Heavy breathing	1	1	1	2	1	2	2	1	1	1	13
Movement	Looking at/playing with hands	0	0	0	0	0	0	0	0	0	0	0
	Lifting head off bed	3	3	3	5	4	2	2	1	2	3	28
	Body jerk	2	0	1	0	1	0	1	1	2	1	9
	Poking out tongue	10	5	7	11	8	6	6	9	2	7	71
Facial expressions	Raising eyebrows	0	0	0	0	0	0	1	1	0	0	2

As illustrated in Table 4, participant 1 engaged in ‘leaning forward to the camera’ (3), ‘feeling her collar’ (1), ‘pointing’ (2), ‘clicking her tongue’ (18) and ‘laughing’ (4) in the Standard sessions but did not do so in the Intervention sessions. However, she engaged in ‘singing’ (11), ‘tutting’ (1), ‘rubbing her knees’ (3), ‘scratching her nose’ (3), ‘pointing’ (2), ‘moving her head from side to side’ (6) and ‘leaning into ME’ (1) during the Adaptive session but did not do so in the Standard session. Participant 1 ‘looked at ME’ over 3 times more often in the Baseline (35) than the Intervention (11) session. However, she also ‘closed her eyes’ twice as much in Baseline (30) than the Intervention (15) session and ‘looked at the camera’ almost twice as many times in the Baseline (27) than Intervention (14) session. The remaining findings saw participant 1 ‘blowing a kiss’ 3 times in Baseline and twice in Intervention session; ‘winking’ 5 times in Standard and 3 times in the Intervention sessions; ‘frowning’ once in Baseline and 4 times in Intervention and ‘smiling’ twice and ‘raising her eyebrows’ 3 times in both types of interaction.

As illustrated in Table 5, participant 2 engaged in ‘pointing’ and ‘shrugging her shoulders’ in the Baseline but did not in the Intervention session. However, she introduced new behaviours in the Intervention that did not appear in the Baseline session, i.e. ‘drawing the ME’s attention’ to sounds outside the room and ‘touching ME’s chin’. The amount of ‘looking at ME’ engaged in by participant 2 was comparable in Baseline (19) and Intervention (21) sessions. Participant 2 ‘laughed’ substantially more often in Intervention (20) than in the Baseline (2) session. This pattern was repeated with regards to ‘moving her hands’ which occurred 35 times in Intervention and only 8 times in the Baseline session; and ‘smiling’ which occurred only once in Baseline but occurred 20 times in the Intervention session. ‘Nodding’ occurred 23 times in Baseline and 17 times in the Intervention session whilst participant 2 ‘shook her head’ 3 times in both types of session. Finally, participant 2 ‘scratched her chin’ once in the Baseline and twice in Intervention session; ‘frowned’ 5 times in the Baseline and 7 times in the Intervention session; and ‘jerked her body’ 4 times in both types of session.

As illustrated in Table 6, there were comparable amounts of ‘looking at ME’ in Baseline (39) and Intervention (35) sessions. Participant 3 engaged in ‘coughing’ (6), ‘yawning’ (2) and ‘smiling’ (1) during the Baseline sessions but did not in the Adaptive sessions. No new behaviours appeared in the Adaptive session for this participant however, there was a marked increase in ‘pointing’ in the Adaptive session (44) as opposed to only one occurrence of this behaviour in the Baseline sessions. There were also almost twice as many ‘speech sounds’ (69, 39) occurrences of ‘hand in air’ (11, 5) in the Adaptive than in the Baseline session. Participant 3 ‘cleared his throat’ 9 times in Baseline and 6 times in Intervention sessions and put his ‘finger in his mouth’ 2 times in the Baseline and 5 times in the Intervention sessions and ‘jerked his body’ 18 times in the Baseline and 7 times in the Intervention session. Finally, participant 3 ‘flashed his eyebrows’ comparable amounts in each types of session with 9 occurrences in the Baseline and 10 in the Intervention session.

Table 7 shows that participant 4 looked at ME 10 times in Baseline and 13 times in the Intervention session. She closed her eyes 8 times in the Baseline and 5 times in the Intervention session. Participant 4 introduced new behaviours in the Adaptive that had not appeared in the Baseline session. She engaged in ‘laughing’ (9), ‘smiling’ (9), showing a facial expression of ‘surprise’ (3) and ‘rubbing her head against the bed’ (6), ‘ME’s hand’ (9) and ‘ME’s head’ (19) in the Adaptive but not the Baseline conditions. She produced her ‘high pitched sound’ 8 times more often in the Adaptive sessions (120) than she did in the Baseline session (15). She also engaged in 3 times more ‘thumb chewing’ in the Baseline (15) than the Adaptive session (5).

As is clear in the Table 8, participant 5 produced ‘speech sounds’ (29), engaged in ‘heavy breathing’ (13) and ‘poked out her tongue’ (71) during the Intervention but did so in the Baseline condition. Furthermore, she ‘looked at and played with her hands’ (16), ‘coughed’ (1), ‘closed her eyes’ (4) and ‘closed her eyes tight’ (2) in the Baseline condition but did not at all during the Intervention condition. Participant 5 ‘looked at ME’ almost 3 times as much in the Intervention (41) than the Baseline (17) interaction session. She ‘lifted her head off the bed’ 11 times in the Baseline and 28 in the Intervention session. Participant 5 ‘jerked her body’ a comparable amount of times in the Baseline (10) and Intervention (9) session. Finally, ‘raising eyebrows’ occurred 11 times more in Baseline (22) than in the Intervention session (2).

### 3. Group comparisons

The communicative behaviours of the nonverbal participants with dementia were compared between conditions using a randomisation test [32]. The nature of this work was exploratory and as such we avoided directional hypotheses. Therefore, both one and two-tailed results were accepted. Table 9 shows the means, standard deviations and p-values for behavioural variables and subvariables between Baseline and Intervention sessions.

**Table 9:** Means, standard deviations (SD) and p-values for behavioural variables and subvariables between Baseline and Intervention sessions.

Variable	Sub variable	Baseline		Intervention		P-values	
		Mean	SD	Mean	SD	One-tailed	Two-tailed
1. Eye gaze	Eyes closed	10.9	19.8	4.7	6	0.78	0.21
	Elsewhere	58.1	32	55.5	37.8	0.38	0.46
	ME’s body/face	**12.6	16.3	**6.2	6	1	0.0004
	Eyes	16.4	18.3	28	28.6	0.11	0.12
	Can’t tell	1.97	7.64	6.32	15.5	0.78	0.78
2. Facial expressions	Neutral	*88.8	25.1	*69.6	33.6	0.97	0.02
	Smiling	***2.8	5	***33.	38.8	4.99	0.0004
	Frowning	0.41	0.85	4.04	14.28	0.13	0.13
	Surprise	0.3	1	0.2	0.8	0.33	0.66
	Other	7.5	25.6	5.8	19.3	0.37	0.37
3. Vocalisations	Silence	78	25	78	15	1	1
	Vocalisation	***18.8	23.9	***18.9	14.9	0.0004	1
	Laughter	0.9	2	2.5	4.2	0.14	0.14
	Other	2.3	4.9	0.5	1	0.37	0.62
4. Physical contact	Contact	0	8.6	0	18.8	1	1
5. Gestures	Pointing	0.6	1.6	0.7	1.6	0.35	0.65
	Nodding	1.9	2.9	0.9	1.4	0.29	0.7
	Shaking head	1.7	3.7	0.5	0.7	0.76	0.23
	Other	7.52	25.65	21.11	34.06	0.43	0.43
6. Imitation	Imitation of ME	*0.1	0.3	*1.7	2.3	0.03	0.03

Means significantly different at

*p < .05

**p < .01

***p < .001

### Communicative behaviours

#### Eye gaze

The randomisation test revealed that people with dementia looked more at ME’s face or body in the Baseline sessions (p < .01). Other variables showed no significant differences.

#### Facial expressions

The randomisation test showed a significantly higher duration of a ‘neutral’ facial expression for people with dementia in Baseline than in Intervention sessions (p < .05; Table 9), where there was a significant increase in ‘smiling’ (p < .01). None of the other facial variables for people with dementia differed significantly.

#### Vocalisations

The randomisation test showed no significant differences in ‘vocalising’ behaviours between Baseline and Intervention sessions (p-values >.05).

#### Bodily contact and gestures

The randomisation test revealed non-significant differences (p-values >.05) in duration of ‘bodily contact’ or type and amount of gestures between Baseline and Intervention sessions for people with dementia.

#### Imitation

There were significant increases in the amount of imitation by people with dementia (p>.05) in Intervention sessions.

## Discussion

### Daily activity

Results of the ‘Direct Observation of Behaviour’ observation instrument [10] indicated that the participants spent the biggest part of the day in a ‘neutral’ state. In other words, for 68.6% of day, the participants were ‘detached from the environment’ and were most often sleeping or doing nothing. This result is particularly striking as it closely resembles the findings of Bowie & Mountain (1993) [10]. One might have expected the social environment for people with advanced with dementia living in care homes to have improved in the last 24 years. However, this does not appear to be true of the care facility participating in this study. ‘Social engagement’ came just below ‘self care’ at 10% indicating that participants spent only one tenth of the waking day in interaction with others. Furthermore it is of interest to note that as in the Bowie & Mountain (1993) [10] study, the majority of these ‘interactions’ involved the caregiver talking to the person with dementia during activities of daily living. This finding suggests that the questions used in the Baseline condition were indeed representative of the interactions that are typically offered by caregivers to individuals with advanced dementia. The next highest percentage was ‘reception of care’ at 5%. This finding largely reflected the level of assistance that the participants required to eat and drink at meal times. ‘Motor activity’ (2%) and so-called ‘inappropriate’ behaviour (2.1%) comprised very little of the day and ‘antisocial’ behaviour did not appear at all. As such, these findings illustrate that very little of the participants’ time was spent engaged in behaviours that might be deemed ‘out of place’ or ‘challenging’. Although this finding is encouraging in itself, it does not reflect the common perception of people with advanced dementia that are held by caregivers; that is they are both unable and unwilling to communicate [6], [13].

### Individual communication repertoires

The use of the Bowie & Mountain (1993) [10] observation instrument not only allowed the observers to sketch out the participants’ daily activity patterns, it also afforded an insight into the communicative behaviours used by the participants. This phase was crucial to the study design as it also allowed the observers to take field notes on and to assess the types of communication engaged in by each individual prior to the intervention, thereby following the principles of Intensive Interaction as defined by Nind (1999) [25]. All participants engaged in a set of fundamental communicative behaviours; eye gaze, sounds, movements and facial expressions, however each individual had his/her own unique subset of behaviours. As such, these were regarded as their individual repertoires.

Participants engaged in different types of communicative behaviours between Baseline and Intervention sessions, i.e. some behaviours increased in Intervention sessions; some behaviours were observed less often in Baseline sessions; some behaviours appeared in each type of session that did not appear in the other. The differences in behaviours between the sessions was sometimes subtle (as with participant 3) and other times very clear (as with participant 4). Indeed, the varieties in response to the Intervention were as individual as the people we worked with. As we have illustrated, each person had his/her own unique vocabulary.

For example, 3 of the participants (1, 2 & 3) retained some form of recognisable speech. These participants responded to the Intervention in a more subtle way than the remaining 2 (4 & 5). What is clear from this finding is that individuals prefer to use the most sophisticated form of communication that they have at their disposal, echoing the ideas of Kitwood [6]. Although these participants did not always use speech to express themselves, they also used their own unique non-verbal methods of communication. For example, participant 1 liked to look at herself in the viewfinder of the camera. She winked at herself and sang in the Intervention session and did not engage in these behaviours in the Baseline session. Participant 2 drew ME’s attention to sounds that were coming from outside her room and touched ME during the Intervention sessions but did not during the Baseline sessions. Furthermore, participant 3 engaged in far more speech sounds and pointing in the Intervention than the Baseline sessions.

### Group comparisons

Each of the five individuals had a communication repertoire and these were distinct from each other. The two individuals (MB & MD) who were the most mobile engaged in more behaviours that were relatively easy to decipher (e.g. pointing, nodding to signify ‘yes’ and shaking the head to signify ‘no’). Only one of the individuals who was confined to bed engaged in one of these, i.e. pointing. However, four of the five participants engaged in making speech sounds that, although difficult to comprehend, indicated a desire to engage in whichever means possible. It also appears that when sounds are less available to the participants, they tend to rely more often on movements and facial expressions to engage. Three of the five participants laughed during the sessions and four of the participants smiled. These findings are particularly encouraging as these behaviours indicate pleasure on the part of the person with dementia and may be easier for caregivers to recognise as signs of emotional connection in the absence of speech [13].

The finding that people with dementia looked more at ‘ME’s body/face’ in Baseline sessions may at first seem to be somewhat anomalous. However, this could be interpreted as the person with dementia ‘scanning’ ME for signs of engagement. The finding that people with dementia displayed a ‘neutral’ facial expression more often in the Baseline than the Adaptive Interaction sessions coupled with significantly more smiling and vocalising, suggests that they enjoyed the nonverbal interactions. Reassuringly as imitation is central to Adaptive Interaction, there was significantly more imitation displayed by the people with dementia. This confirms that imitation may provide a feasible method for nonverbal people living with dementia to communicate with others.

### General discussion

In interpreting these findings we must of course look to the theory behind Intensive/Adaptive Interaction. Viewed from the developmental perspective, we can assume that ME’s imitation of the person with dementia’s communicative behaviours provided him/her with meaningful feedback. This feedback encouraged the person with dementia to further engage with ME and as such a reciprocal interaction ensued. We can also claim that ME’s use of Adaptive Interaction unlocked a wider communicative repertoire in people with advanced dementia in comparison to Baseline sessions. Adaptive Interaction revealed new behaviours in individuals with advanced dementia that Baseline Interactions did not. These findings were perhaps most clear in the individuals who have the most advanced dementia. For example, although EA and GB were probably the most cognitively impaired, the use of their own communicative behaviours by ME appeared to have a hugely positive effect. GB laughed a total of 9 times in the Intervention sessions and did not laugh at all in the Baseline sessions. The presence of laughter as a communicative behaviour in this context does not require a lengthy interpretation or theoretical supposition. As such, we can surmise that participant 4 simply enjoyed the Intervention session more and found it more fun and amusing than the Baseline session. Participant 5’s reaction to ME’s use of Adaptive Interaction was also extremely noteworthy. For example, she did not produce speech sounds or engage in heavy breathing at any point during the Baseline condition but did so in the Intervention condition (n = 29). The presence of these behaviours in the Intervention condition indicates that participant 5 made far more attempts to communicate with ME. Indeed, increased eye contact with ME supports the claim that her desire to communicate was much clearer in the Intervention condition. ME’s behaviour, i.e. a reflection and interpretation of her own communicative repertoire, not only caught her interest but was also meaningful to her. Participant 5 appeared far more interested in herself and her own immediate surroundings during the Baseline than Intervention sessions. This was illustrated by her looking at and playing with her hands in Baseline sessions (n = 16). When she did this, her hands were positioned directly in front of her face and she appeared fascinated by the movement of her fingers. This is reminiscent of the ‘self-talk’ behaviours that people with Autistic Spectrum Disorders and severe and profound learning disabilities may engage in [9]. Participant 5 did not engage in this ‘self-talk’ behaviour at any point during the Intervention condition. Instead she looked directly at ME with more regularity than in the Baseline condition and the presence of speech sounds in the Intervention condition indicated a higher level of engagement than in the Baseline condition.

From this small-n study, Adaptive Interaction appears to have potential for promoting and supporting communication between people living with dementia who cannot speak and those who care for them. This is a hopeful finding for this population as they typically have few opportunities for social participation in their environment. Similar to the observations of Bowie and Mountain [10] more than 20 years ago, more than two thirds of their time was spent engaged in no activity whatsoever. ‘Social engagement’ accounted for just 10% of the day, with the majority of these ‘interactions’ consisting of a caregiver speaking to a person with dementia whilst assisting them with or carrying out activities of daily living. They were offered little or no stimulation, social interaction or meaningful engagement, in accordance with the mistaken perception that people living with dementia without verbal language production are ‘unreachable’.

Overall, these findings suggest that Adaptive Interaction provides a mechanism for people living with dementia who have no functional verbal language production, to demonstrate a desire and ability to communicate. The study highlights that interaction partners need to be responsive and adaptive to the needs of nonverbal people with dementia. Providing a supportive communication environment can enable nonverbal people with dementia to demonstrate their continued personhood in social interactions with another human being.

This study has shown that, certainly in the participating care home, people with very advanced dementia have very few opportunities for social interaction. The data gleaned by the Bowie & Mountain [10] observation instrument in this study closely reflected those reported in the original study. This finding is somewhat counterintuitive as we would expect it to be rather more positive in response to today’s supposedly enlightened approach to dementia care. However, the results of the current study have also shown that it is possible to both identify and use the individual communicative repertoires of people with advanced dementia and that Adaptive Interaction has a positive impact on their communicative abilities. This illustrates that contrary to popular belief, these individuals although severely cognitively impaired and communicatively challenged have both the desire and the means to communicate with other individuals. The biggest challenge in this study was to identify and use individual repertoires in a way that were meaningful to each person. Were the Adaptive Interaction approach to be employed within dementia care environments, the bigger test would be to convince care staff of the validity of this approach. Furthermore, it is likely that training staff in how to engage in Adaptive Interaction would in itself present the most significant obstacle. However, we hope that any initial feelings of self-consciousness on the part of care staff will be replaced with pride in response to what they and the person they are working with achieve together. With this in mind we plan to take Adaptive Interaction further by training a small group of dementia care home staff to use the approach. The wider aim thereafter will be to roll out this approach in care homes across the country. This in turn will hopefully go some way to changing the patterns of and opportunities for social interactions in care homes for the better.

### Limitations and future directions

Perhaps the main criticism that may be levelled at this study is the appropriateness of having a single individual acting as the sole interaction partner to all participants. As such, one might question the potential generalisation of the results in situations where other communication partners are involved. A single communication partner was used for three main reasons. First of all, ME is well versed in facilitative communication strategies and is aware of and sensitive to the communicative needs of people with dementia. Secondly, this design provides a means of exerting a modest amount of control over the findings. In short, all participants engaged with one individual thereby ruling out the impact of differing knowledge bases and approaches that may have been used by other interaction partners. Thirdly, training other interaction partners to engage with people with dementia using Adaptive Interaction was out with the scope of this study. However, as previously mentioned this is something that we aim to explore in our future research.

## Supporting information

S1 File Example of Adaptive interaction. (DOCX)

S2 File Baseline interaction questions. (DOCX)
