# Correction: Communicating with people living with dementia who are nonverbal: The creation of Adaptive Interaction

**DOI:** 10.1371/journal.pone.0196489

**Published:** 2018-04-23

**Authors:** Maggie Ellis, Arlene Astell

One point has been amended in the raw data file, and the authors have uploaded the corrected data set under a different figshare DOI. The correct Data Availability statement is: Data are available from figshare (DOI: https://doi.org/10.6084/m9.figshare.5398312). Data are from the Adaptive Interaction study whose authors may be contacted at the University of St Andrews: mpe2@st-andrews.ac.uk.

There is an error in the third sentence in the paragraph titled “3. Group comparisons” under the Results section. The correct sentence should read: Therefore, two-tailed results were accepted.

There is an error in the sentence under the subheading “Imitation” in the paragraph titled “Communicative behaviours” under the Results section. The correct sentence should read: There were significant increases in the amount of imitation by people with dementia (p < .05) in Intervention sessions.

There are multiple errors in Table 2. The subvariables Looking elsewhere, Looking at ME’s face or body, Other (e.g. coughing), Person with dementia imitates ME should be modified for clarity. The subvariables Eyes and Other expression should be added. The subvariables No contact and Can’t tell should be removed.

**Table 2:** The main behavioural coding categories and subvariables.

Coding category	Subvariable
1. Eye gaze	Eyes closed
	Elsewhere
	Partner’s body/face
	Eyes
	Can’t tell
2. Facial expressions	Neutral
	Smiling
	Frowning
	Surprise
	Other expression
3. Vocalisations	Silence
	Vocalisations
	Laughter
	Other vocalisation (e.g. coughing)
4. Physical contact	Contact occurs
5. Gestures	Pointing
	Nodding
	Shaking head
	Other gesture
6. Imitation	Imitation of partner

There are multiple errors in Table 9 and its caption. The values in Eye Gaze: Partner’s body/face: Two-tailed has been corrected to <0.001. The value in Facial expressions: Neutral: Two-tailed has been corrected to 0.027. The value in Facial Expressions: Smiling: Two-tailed has been corrected to <0.001. The value in Imitation: Imitation of partner: Two-tailed has been corrected to 0.039. In the Table 9 caption, the text “p-values for behavioural variables and subvariables between Baseline and Intervention sessions” should be changed to “*two-tailed p-values for behavioural variables and subvariables of 3 minute analysis periods within Baseline and Intervention sessions.”

**Table 9:** Means, standard deviations (SD) and *two-tailed p-values for behavioural variables and subvariables of 3 minute analysis periods within Baseline and Intervention sessions.

Variable	Sub variable	Baseline		Intervention	
		Mean	SD	Mean	SD	Two-tailed
1. Eye gaze *(% of total time sampled)*	Eyes closed	11.34	10.62	3.98	4.30	0.214
	Elsewhere	54.52	24.86	59.98	37.16	0.460
	Partner’s body/face	13.58	10.73	6.14	4.26	**<0.001**
	Eyes	18.56	18.53	25.91	25.17	0.122
	Can’t tell	1.97	4.41	4.74	10.59	0.780
2. Facial expressions *(% of total time sampled)*	Neutral	88.52	12.68	74.09	28.67	**0.027**
	Smiling	2.85	3.95	10.24	9.13	**<0.001**
	Frowning	0.38	0.36	4.04	8.12	0.139
	Surprise	0.42	0.93	0.20	0.44	0.667
	Other expression	7.69	14.38	11.43	19.63	0.732
3. Vocalisations *(% of total time sampled)*	Silence	78.56	20.44	79.45	11.32	1.000
	Vocalisation	18.65	19.57	17.60	11.20	1.000
	Laughter	0.87	1.42	2.26	3.35	0.146
	Other vocalisation	1.91	2.66	0.65	0.97	0.625
4. Physical contact *(% of total time sampled)*	Contact	0.00	0.00	7.09	10.44	1.000
5. Gestures *(Number of occurrences within time sampled)*	Pointing	0.60	1.16	0.86	1.23	0.654
	Nodding	1.90	2.45	0.91	1.15	0.700
	Shaking head	1.73	3.34	0.46	0.64	0.234
	Other gesture	7.69	14.36	21.42	24.48	0.431
6. Imitation *(Number of occurrences within time sampled)*	Imitation of partner	0.11	0.16	1.43	1.55	**0.039**

*Analysis conducted using AB multiple baseline randomisation test macro provided by Todman & Dugard (2001).

Please see the complete, correct Tables 2 and 9 here.
