# Supplementary material for: Communicating with people living with dementia who are nonverbal: The creation of Adaptive Interaction
Source: PLoS One. 2017 Aug 1;12(8):e0180395. doi: 10.1371/journal.pone.0180395 (PMC5538738; doi:10.1371/journal.pone.0180395)
Supplement: S1 File — (DOCX) [file pone.0180395.s001.docx]

Appendix

The following passage represents an example of an Adaptive Interaction session between the first author (MPE) and Edie, first reported in: Ellis MP, Astell AJ. Promoting communication with people with severe dementia. S. Zeedyk (Ed). Techniques for promoting social engagement in individuals with communicative impairments. Jessica Kingsley Publishers, 2008.

At the start of this session Edie was lying in her bed with padded cot-sides. She was lying on her side on two pillows, dozing. After 16 seconds Edie opened her eyes and looked directly at MPE and made ‘her’ sound in a high-pitched tone. MPE immediately reflected the sound and pitch back to her. Edie then repeated the sound and both interaction partners took another 2 turns each in this manner. Edie’s immediate reaction to MPE speaking was to look at her and make the high-pitched sound. MPE adapted her response to match Edie’s, which resulted in a brief initial ‘dialogue’ of several turns each.

This phase of the session was notable for Edie introducing touch into the interaction. The dialogue had been proceeding through sound and vision (eye contact) when Edie opened up a third channel of communication, i.e. touch. However, although MPE responded by touching Edie’s head, MPE did not match her behaviour, as she had done with Edie’s sound turn-taking. Edie then rubbed her head against MPE’s hand for a third time and MPE moved forward and rubbed her head against Edie’s. At this point, Edie opened her eyes and gave a look of surprise followed by the high-pitched sound. The dialogue then took on the form of a spontaneous game of mutual head touching and vocalisation. During this phase Edie laughed at several points after she and MPE touched heads. This is perhaps the most exciting part of the interaction as this is when Edie exerted the most control over the situation and was the most animated. Edie was clearly attempting to get closer to MPE and to touch her. However, initially MPE failed to detect this and was focused on maintaining previous strategies of the interaction. Once the MPE recognised Edie’s new direction, the interaction took on a new dynamic. From the moment MPE touched heads with Edie, their communication became much more playful. They took turns with sounds and touching and both laughed at several points throughout.

At 7 minutes and 4 seconds into the interaction, Edie fell silent and closed her eyes. She remained like this until MPE touched her head 46 seconds later, at which point she made her sound and then opened her eyes when MPE reciprocated with the sound. Edie and MPE began turn-taking again using Edie’s sound and both laughed several times. At 9 minutes and 1 second, Edie fell silent and then closed her eyes 5 seconds later. She remained like this for the rest of the session. This section suggests that perhaps Edie was ready to end the interaction at a point before MPE realised. MPE attempted to keep the interaction going and Edie reciprocated with enthusiasm for a while but closed her eyes again very soon after. Edie closing her eyes effectively ended the interaction and can be seen as another element of her communication repertoire.

At the start of both sessions Edie made eye contact with MPE and a high-pitched sound. In the SI Session, however, Edie quickly stopped making any sound or eye contact and at 67 seconds into the 10 minutes, effectively disengaged from the interaction. By contrast in the II Session, MPE’s reciprocation of Edie’s initial communication bids, led to turn-taking and a rather more intimate interaction.

Instead of continuing to speak, MPE adapted her response to match Edie’s, which resulted in a brief initial ‘dialogue’ of several turns each. At 23 seconds into the interaction, the dialogue changed when Edie put her thumb in her mouth and started sucking and chewing on it, all the time looking into MPE’s eyes. MPE responded by sucking and chewing her own thumb. Edie then removed her thumb from her mouth and made her high-pitched sound. MPE responded by taking her own thumb from her mouth and repeating the sound made by Edie. Edie then put her thumb back into her mouth, and MPE followed suit. In these exchanges Edie took the lead by introducing a new behaviour (thumb-chewing), then reverting to the previous behaviour (high-pitched sound) then returning to thumb chewing, all the while looking intently at MPE. MPE responded to each of these changes by matching Edie’s behaviour.

MPE then attempted to change the dialogue by removing her own thumb from her mouth and making a sound like Edie’s. In response Edie then removed her thumb from her mouth and matched the sound and she and MPE then continued to turn-take making this sound for another 20 seconds. This section of dialogue ended when Edie began sucking her thumb again. In this exchange MPE reintroduced one of Edie’s behaviours (high-pitched sound) and Edie responded by altering her own behaviour to match hers.

At 90 seconds into the 10-minute session, MPE attempted to change the interaction again by introducing a new element. This was to imitate the rhythm of Edie’s thumb chewing through tapping her own fingers on the side of the bed. Edie continued to chew her thumb and stared intently at MPE. After a few seconds, Edie removed her thumb from her mouth and made her high-pitched sound. MPE stopped tapping and repeated the vocal sound and turn-taking resumed using Edie’s sound until MPE tapped on the bed again. Edie became silent, put her thumb back in her mouth and watched MPE’s fingers tapping on the bed. Edie then removed her thumb from her mouth and resumed her high-pitched sound. At 108 seconds, Edie put her thumb in her mouth and immediately removed it when she saw me do the same. Edie and MPE then resumed turn taking with her sound.

In this phase, when MPE introduced the new element (rhythmic tapping) there was no discernible change in Edie’s behaviour. She continued to chew her thumb while looking intently at MPE. However, as MPE continued to tap, Edie then stopped chewing and made her high-pitched sound. She did not put her thumb in her mouth again during this session. The turns in this exchange suggest that the introduction of a variation of one her behaviours (thumb-chewing) had less impact for Edie than the matched behaviour. However, she appeared to retain her interest in the interaction as she continued to look at MPE and finally reintroduced a previous behaviour (high-pitched sound).

Edie and MPE continued the dialogue making the high-pitched sound until 150 seconds into the session, at which point Edie introduced another new behaviour. She lifted her head up from the pillows and moved towards MPE’s hand, which was resting on the cot-side. Edie rubbed her forehead on MPE’s hand and MPE responded by stroking Edie’s hair. MPE then attempted to reintroduce one of Edie’s previous behaviours, i.e. her thumb-sucking and the rhythm of it. Again, Edie raised her head, rubbed her forehead against MPE’s hand and then closed her eyes. MPE then made Edie’s sound towards her to which she reciprocated followed by a number of turns each. Edie continued to keep her eyes closed for 43 seconds during this part of the interaction.
