# Supplementary material for: Communicating with people living with dementia who are nonverbal: The creation of Adaptive Interaction
Source: PLoS One. 2017 Aug 1;12(8):e0180395. doi: 10.1371/journal.pone.0180395 (PMC5538738; doi:10.1371/journal.pone.0180395)
Supplement: S2 File — (DOCX) [file pone.0180395.s002.docx]

**Baseline interaction questions**

- Hello (participant’s name), I’m (Investigator’s name). Would you like to have

a chat with me?

- Are you well today?
- Have you had breakfast/lunch/dinner?
- Did you enjoy your meal?
- Have you had a good day so far?
- Did you sleep well last night?
- Did you go to bed early?
- Did you have a lie in this morning?
- Have you taken part in any activities today?
- Do you usually take part in the activities?
- Do you enjoy the activities here?
- Have you seen the news today?
- Did you see the weather report?
- Have you seen the weather outside today?
- It’s lovely/miserable, isn’t it?
- It’s looking more like spring/summer/autumn/winter now, isn’t it?
- It’ll soon be spring/summer/autumn/winter won’t it?
- Are you looking forward to it?
- Well, thanks for your time. It was nice talking to you.
- I have to go now. I’ll see you again soon. Bye.

**Baseline interaction questions**

- Hello (participant’s name), I’m (Investigator’s name). Would you like to have

a chat with me?

- Are you well today?
- Have you had breakfast/lunch/dinner?
- Did you enjoy your meal?
- Have you had a good day so far?
- Did you sleep well last night?
- Did you go to bed early?
- Did you have a lie in this morning?
- Have you taken part in any activities today?
- Do you usually take part in the activities?
- Do you enjoy the activities here?
- Have you seen the news today?
- Did you see the weather report?
- Have you seen the weather outside today?
- It’s lovely/miserable, isn’t it?
- It’s looking more like spring/summer/autumn/winter now, isn’t it?
- It’ll soon be spring/summer/autumn/winter won’t it?
- Are you looking forward to it?
- Well, thanks for your time. It was nice talking to you.
- I have to go now. I’ll see you again soon. Bye.
